# Supplementary figures and images for: Chitosan Oligosaccharides Coupling Inhibits Bacterial Biofilm-Related Antibiotic Resistance against Florfenicol
Source: Molecules. 2020 Dec 21;25(24):6043. doi: 10.3390/molecules25246043 (PMC7767115; doi:10.3390/molecules25246043)

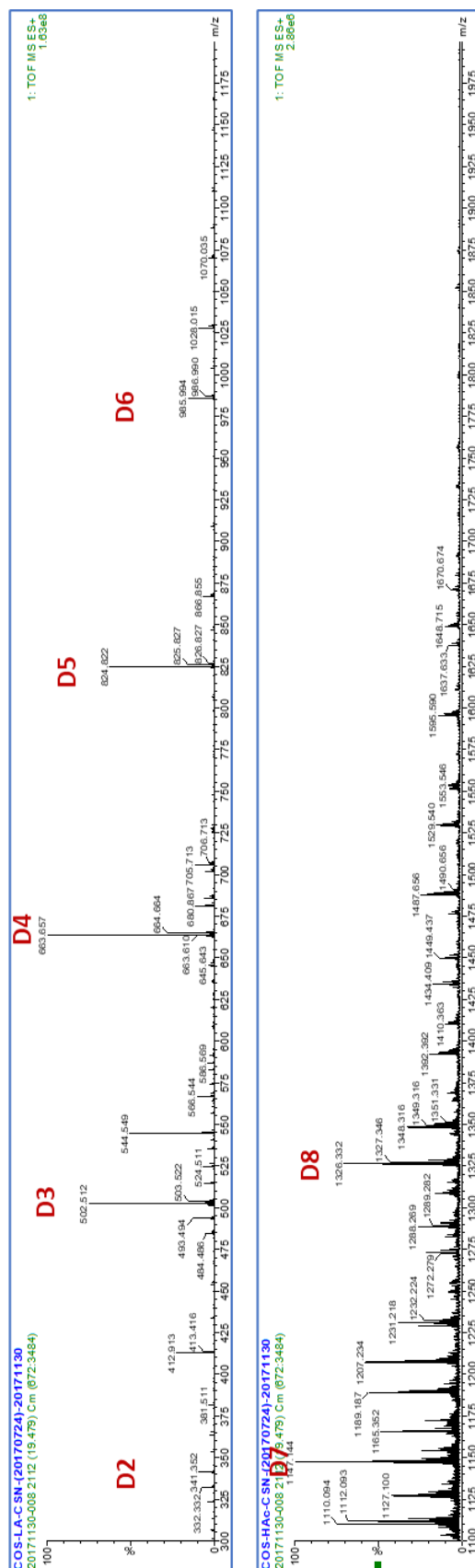

**Figure S1.** The characterization of COS by UPLC-Q TOF MS spectrometry in the positive ion mode.

Supplement: Supplementary file 1 [file molecules-25-06043-s001.pdf]
